# Adjustment of branched-chain amino acid balance fails to prevent feed intake decline in lactating sows fed high soybean meal diets

**DOI:** 10.1093/jas/skaf072

**Published:** 2025-03-06

**Authors:** Dalton C Humphrey, Laura L Greiner

**Affiliations:** Department of Animal Science, Iowa State University, Ames, IA, USA; Department of Animal Science, Iowa State University, Ames, IA, USA

**Keywords:** branched-chain amino acids, feed intake, lactation, soybean meal, sows

## Abstract

A total of 352 sows were used to investigate the effect of soybean meal (**SBM**) level and adjusted branched-chain amino acid (**BCAA**) balance on lactating sow and piglet performance. On day 112 ± 1.5 of gestation, sows were randomly assigned, within parity, to one of four dietary treatments: low SBM (**LSBM**), high SBM (**HSBM**), LSBM with adjusted BCAA (LSBM + BCAA), or HSBM with adjusted BCAA (HSBM + BCAA). The BCAA balance was adjusted to achieve equal standardized ileal digestible (**SID**) Ile:Leu:Val in the LSBM and HSBM + BCAA diets (SID Ile:Leu:Val 0.49:1.00:0.73) and in the HSBM and LSBM + BCAA diets (SID Ile:Leu:Val 0.54:1.00:0.58). All diets were formulated to be equal in SID Lys, isocaloric, and meet or exceed NRC (NRC. 2012. Nutrient requirements of swine. 11th rev. ed. Washington (DC). Natl. Acad. Press) recommendations for all other essential amino acids, vitamins, and minerals. Sow body weight (**BW**) and backfat thickness (**BF**) were measured at the time of entry into the farrowing room and at weaning. Litter weights were captured after cross-fostering and at weaning to calculate litter growth rate. Data were analyzed using generalized linear mixed models in R (v4.4.1; R Core Team, R Core Team. 2024. R: a language and environment for statistical computing. Vienna (Austria): R Foundation for Statistical Computing. https://www.R-project.org/) with fixed effects of dietary treatment, parity group, and their interaction and a random effect of lactation group. The sow and her litter were the experimental unit, and results were considered significant if *P* ≤ 0.05. There was an effect of the dietary treatments on sow average daily feed intake (ADFI; *P* < 0.001). Sows consuming the LSBM diet exhibited greater ADFI compared to HSBM (7.43 vs. 7.05 kg/d; *P* = 0.024) or HSBM + BCAA (7.43 vs. 6.87; *P* < 0.001), while sows fed LSBM + BCAA showed greater ADFI than HSBM + BCAA (7.24 vs. 6.87; *P* = 0.025), but similar ADFI to LSBM- (*P* = 0.515) and HSBM-fed (*P* = 0.466) fed sows. Despite differences in ADFI, sows gained an average of 7.1 kg of BW (*P* = 0.682) and lost 1.3 mm of BF (*P* = 0.928) through the lactation period. Sows started the trial with an average of 14.0 piglets/sow (*P* = 0.787) and weaned 12.6 piglets/sow (*P* = 0.875) with a piglet average daily gain of 0.22 kg/d (*P* = 0.646). Increasing SBM inclusion by 14% reduced sow ADFI in lactation by approximately 5% but did not alter sow BW or BF loss or piglet growth rate. While adjusting BCAA in the LSBM diet slightly reduced sow feed intake, adjusting BCAA in the HSBM diet did not alleviate the reduction in ADFI caused by elevated SBM, suggesting the balance of BCAA does not contribute to the feed intake response observed with elevated SBM inclusion in lactation diets.

## Teaser

Balancing the branched-chain amino acid balance did not mitigate the reduction in sow lactation feed intake observed with feeding diets containing elevated levels of soybean meal.

## Introduction

Lactation is the most metabolically demanding period in the reproductive lifetime of a sow, and maximizing feed intake during this time is crucial to ensure adequate intake of nutrients to support milk production and minimize maternal tissue mobilization, as it is well documented that excessive tissue mobilization during lactation reduces milk yield and subsequent reproductive performance (Koketsu et al., 1996). Therefore, it is imperative that the composition of the diet is not only adequate in terms of nutrient concentration relative to the requirements of the sow but also that the ingredients that constitute the diet do not adversely affect feed intake.

Soybean meal (**SBM**) represents a valuable amino acid source in swine diets due to the high level and balance of digestible essential amino acids, which complement the amino acid profiles of common cereal grains, such as corn. It is common for nutritionists to limit the inclusion of SBM in sow lactation diets due to observations of reduced feed intake at elevated levels of SBM (Yang et al., 2000a; Gourley et al., 2020; Estrada et al., 2024; PIC North America, 2024).

The reduction in feed intake associated with elevated levels of SBM in the diet has been speculated to be caused by imbalances of the branched-chain amino acids (**BCAA**) leucine, isoleucine, and valine (Yang et al., 2000a; Gourley et al., 2020). The first two enzymes in BCAA catabolism are shared amongst the three amino acids; therefore, feeding an excess level of one BCAA will result in the catabolism of all three, which may result in inadequate availability of the BCAA not fed in excess (D’Mello, 2003; Brosnan and Brosnan, 2006). Consequently, an induced deficiency of a BCAA caused by elevated catabolism may trigger a hypophagic response, which is a well-documented phenotype associated with amino acid deficiency or imbalance (Harper et al., 1970).

The NRC (2012) estimated standardized ileal digestible (**SID**) Ile:Lys, Leu:Lys, and Val:Lys requirements for lactating sows are approximately 56%, 113%, and 85%, respectively, corresponding to a SID Ile:Leu:Val of 0.50:1.00:0.75. It is commonly economical to include feed-grade L-Val in sow lactation diets, while SID Ile and Leu needs can be readily met or exceeded with intact protein sources, such as SBM. Dehulled solvent-extracted SBM contains a SID Ile:Leu:Val of 0.61:1.00:0.61 (NRC, 2012); thus, relative to the sow’s requirements, SBM contains disproportionally high levels of Ile and Leu compared to Val. Therefore, increasing SBM inclusion will displace L-Val, exceed Ile and Leu requirements, and potentially create an imbalance between the BCAA, which could result in reduced sow performance.

No research has been conducted to understand if adjusting the BCAA balance in diets containing elevated SBM levels will alleviate the potential negative impacts on feed intake and the pursuant increase in sow body tissue mobilization. Therefore, the objective of this study was to evaluate if the relative balance of Leu, Ile, and Val contributes to the previously observed reduction in sow feed intake with elevated SBM inclusion and to determine if adjusting the BCAA levels in the diet alleviates the response. The hypothesis was that increasing SBM inclusion relative to a standard corn-SBM diet with feed-grade amino acids would reduce sow feed intake and result in increased BW loss in lactation, but adjusting the SID Ile:Leu:Val to that of the standard diet would alleviate these effects. Additionally, it was hypothesized that adjusting the SID Ile:Leu:Val in the standard diet to that of the high SBM diet would result in similar reductions in feed intake and BW loss to the high SBM diet.

## Materials and Methods

### General

All experimental protocols adhered to guidelines for the ethical and humane use of animals for research according to the Guide for the Care and Use of Agricultural Animals in Research and Teaching (FASS, 2010) and were approved by the Institutional Animal Care and Use Committee at Iowa State University (IACUC 23-200).

### Animals

The study was conducted from January through February 2024 at a Midwestern commercial sow research facility. A total of 352 primiparous and multiparous sows (PIC Camborough; PIC Genus, Hendersonville, TN) bred to terminal line boars (PIC 800; PIC Genus, Hendersonville, TN) were utilized across five lactation groups. The sow herd was porcine reproductive and respiratory syndrome virus stable. The sows were fed a standard gestation diet according to body condition score (**BCS**; 4.1, 3.4, and 2.9 kg for BCS 2, 3, and 4, respectively). The gestation diet contained 3.10 Mcal ME/kg and 0.61% SID Lys. At the time of entry into the farrowing room, sows were randomly assigned, within parity, to one of four dietary treatments containing high or low SBM with or without adjusted BCAA levels.

### Experimental diets

The experimental diets (Table 1) consisted of a low SBM diet (LSBM), which contained the minimum level of SBM needed to meet all essential amino acid requirements without adding L-Ile, and a high SBM diet (HSBM) that included the minimum level of SBM required to fully replace L-Lys, resulting in a 14% increase in SBM inclusion and 4.5% increase in crude protein (CP) in the HSBM compared to the LSBM diet. The BCAA ratios in the LSBM diet were then adjusted (LSBM + BCAA) to achieve a SID Ile:Leu:Val equal to the HSBM diet (SID Ile:Leu:Val 0.54:1.00:0.58). Similarly, the BCAA ratios in the HSBM diet were adjusted (HSBM + BCAA) to be equal to the LSBM diet (SID Ile:Leu:Val 0.49:1.00:0.73). The LSBM + BCAA and HSBM + BCAA diets were achieved by replacing cornstarch with L-Leu, L-Ile, and L-Val in the LSBM and HSBM diets, respectively. SID Lys (1.10%) was held constant, and diets were formulated to be isocaloric (3.20 Mcal ME/kg) by adding fat to the HSBM and HSBM + BCAA diets. Other feed-grade amino acids (Met, Thr, Trp) were added as needed to achieve equal levels relative to Lys across all diets. Additionally, diets were formulated to meet or exceed all essential amino acid and vitamin requirements based on NRC (2012) recommendations, and calcium and available phosphorus levels were equal across diets.

**Table 1.** Ingredient and calculated nutrient composition of experimental diets (as-fed basis)

	Dietary treatment			
Ingredient	LSBM	HSBM	LSBM + BCAA	HSBM + BCAA
Corn	70.30	57.42	70.30	57.42
Soybean meal 46.5% CP	23.26	37.26	23.26	37.26
Calcium carbonate	1.06	0.98	1.06	0.98
Monocalcium phosphate	1.09	1.00	1.09	1.00
Fat AV blend	1.00	1.45	1.00	1.45
Corn starch	0.64	0.59	–	–
Sodium chloride	0.50	0.50	0.50	0.50
Other feed additives^1^	0.45	0.45	0.45	0.45
Liquid lysine 55%	0.63	–	0.63	-
ThrPro^2^	0.28	0.03	0.28	0.03
VTM premix with phytase^3^^,^^4^	0.15	0.15	0.15	0.15
HMTBa^5^	0.17	0.02	0.17	0.02
Choline chloride	0.12	0.12	0.12	0.12
L-tryptophan	0.07	–	0.07	–
25-hydroxyvitamin D_3_	0.03	0.03	0.03	0.03
L-isoleucine	–	–	0.29	–
L-leucine	–	–	0.35	0.19
L-valine	0.26	–	0.26	0.40
Total	100.00	100.00	100.00	100.00
Calculated composition				
ME, kcal/kg	3.20	3.20	3.20	3.20
Crude protein, %	16.73	21.30	17.00	21.61
Crude fat, %	3.78	4.01	3.78	4.01
Calcium, %	0.85	0.85	0.85	0.85
Available phosphorus, %	0.44	0.44	0.44	0.44
Neutral detergent fiber, %	6.40	6.46	6.40	6.46
Acid detergent fiber, %	1.49	1.76	1.49	1.76
Total Lys, %	1.24	1.29	1.24	1.29
Total Ile, %	0.70	0.99	0.96	0.99
Total Leu, %	1.47	1.87	1.81	2.06
Total Val, %	0.78	1.06	1.13	1.25
SID Lys, %	1.10	1.10	1.10	1.10
SID Ile, %	0.62	0.87	0.87	0.87
SID Leu, %	1.27	1.61	1.61	1.80
SID Val, %	0.93	0.93	0.93	1.33
SID TBCAA, %	2.82	3.42	3.42	4.00
SID Arg:Lys, %	0.92	1.31	0.92	1.31
SID His:Lys	0.35	0.46	0.35	0.46
SID Ile:Lys	0.56	0.80	0.80	0.80
SID Leu:Lys	1.15	1.47	1.47	1.64
SID Met + Cys:Lys	0.53	0.53	0.53	0.53
SID Phe:Lys	0.65	0.87	0.65	0.87
SID Thr:Lys	0.64	0.64	0.64	0.64
SID Trp:Lys	0.22	0.22	0.22	0.22
SID Tyr:Lys	0.40	0.53	0.40	0.53
SID Val:Lys	0.85	0.85	0.85	1.21
SID TBCAA:Lys	2.56	3.11	3.11	3.64
SID Ile:Leu	0.49	0.54	0.54	0.49
SID Val:Leu	0.74	0.58	0.58	0.74
SID Ile:Val	0.66	0.94	0.94	0.66

^1^Included 0.40% Vigilex (Provimi North America) and 0.05% Promote Diffusion 501 (Provimi North America).

^2^Threonine biomass product (CJ America – Bio) provided 80% L-threonine.

^3^Vitamin trace mineral premix; Provided 9,011 IU vitamin A, 2,254 IU Vitamin D, 60 IU vitamin E, 3.8 mg vitamin K, 7.1 mg riboflavin, 39.9 mg niacin, 2.2 mg thiamin, 1.7 mg folic acid, 0.2 mg biotin, 20 mg Cu (copper sulfate and basic copper chloride), 0.7 mg I (ethylenediamine dihydroiodide), 100 mg Fe (ferrous sulfate), 50.1 mg Mn (manganous oxide), 0.3 mg Se (sodium selenite and selenomethionine hydroxy analogue), and 120 mg Zn (zinc sulfate) per kg of the diet.

^4^Phytase provided at 978 FTU per kg of feed, assuming 0.15% available phosphorus release.

^5^HMTBa; Methionine source. 88% 2-hydroxy,4-methylthio butanoic acid.

The diets were manufactured at a local feed mill. Prior to diet formulation, SID amino acid concentrations in corn and SBM samples from the feed mill were estimated using near-infrared spectroscopy (NIR). Furthermore, the SBM samples were submitted to the University of Missouri Agricultural Experimental Station Laboratories (Columbia, MO) for complete amino acid profiling to validate the NIR amino acid estimates. Feed samples were collected weekly and stored at −20 °C until further analysis.

### Lactation management and feeding

Sows were moved to the farrowing room at 112 ± 1.5 d of gestation and were fed their assigned dietary treatments from entry until weaning at 20 ± 1.2 d of lactation. The sows were housed in standard farrowing stalls (1.5 × 2.1 m) in an environmentally controlled farrowing room (19 to 24 °C). Sows farrowed at 117 ± 1.4 d of gestation, and piglets were cross-fostered, within treatment, within 24 h after birth. Piglets were processed on day three of age, which consisted of docking tails, 200 mg injection of iron dextran, and surgical castration of male piglets.

Upon entering the farrowing room, sows were fed 2.18 kg/d of their allocated lactation diet until parturition. After farrowing, sows were fed 4.35 kg on day one, 6.53 kg on day two, 8.70 kg on day three postpartum, and then allowed ad libitum access to feed for the remainder of lactation. Accordingly, feed was hand-delivered to feed hoppers (Liberty Feeder; Thorp Equipment Inc., Thorp, WI) twice daily, with excess feed removed and weighed as needed. Sows and piglets had ad libitum access to water throughout lactation.

### Sow and litter criteria

Sow body weight (**BW**) was recorded at entry into the farrowing room and at weaning. Sow 48h postfarrowing BW was calculated according to the following empirical equation: $post-farrow\;weight,\;kg\;=\;\left[\left(d112\;gestation\;weight,\;kg\times \;0.98\right)-\;20.81\;\left(R\hat{\;}2\;=\;0.93\right)\right]$ (Greiner, unpublished data). The equation used for estimating sow 48h postfarrowing BW was developed using sows of a similar genetic background, weight range, and production capacity as the sows in the current study. Additionally, sow backfat (BF) at the last rib was measured by ultrasound (Tecnoscan SF-1; Tecnovet, Barcelona, Spain) at the time of entry into the farrowing room and at weaning. The sow metabolizable energy (**ME**) requirement for maintenance (ME_m_) and the energy secreted in milk were calculated according to the following equations:

$ME\_\left(m\right),\;kcal/d=110\;kcal\times BW\hat{\;}0.75$ (Dourmad et al., 2008; NRC, 2012)

Energy in milk, kcal/d= [(4.92 × LGR) (90×LS)]    (NRC, 2012)

Where BW is the calculated sow 48h postfarrowing BW, LGR is the litter average daily gain (**ADG**), and LS is the average number of pigs per litter. The ME required for energy secreted in milk (ME_l_) was then calculated assuming an efficiency of conversion of ME to milk energy of 0.78 (Theil et al., 2004). The ME balance of the sow was considered the difference between ME intake and the sum of ME_m_ and the ME required for milk.

After weaning, sows were returned to the standard gestation diet and fed according to BCS. Sows were checked daily for signs of estrus using a mature boar starting three days postweaning, and estrus was recorded when sows stood to be mounted by a boar. Wean-to-estrus interval (**WEI**) and subsequent total born, born alive, stillborns, and mummies were recorded. Only sows bred within 14 days postweaning were used to collect WEI and subsequent reproductive performance data. Additionally, sows identified to be culled after weaning were not included in WEI or subsequent performance.

Piglet weights were recorded within 24 h of birth and at weaning. Cross-fostering was conducted within treatment, within 24 h of birth, to equalize litter size across treatments. Any piglet mortalities or morbidities were recorded along with the weights of the removed piglets. The weights and nursing days of the removed piglets were back-calculated into litter growth rate using the following equation: $\begin{array}{l} \text{Piglet ADG},\text{kg}/\text{d}=(\text{total litter wean weight}-\text{total starting litter weight]}\\ +\text{removal weights})/[(\text{number of piglets weaned}\times \text{lactation length})\\ +\text{days removals nursed}.\text{]} \end{array}$ Similarly, litter growth rate was calculated using the following simplified equation: Litter ​​​ ADG,kg/d=(total ​​ ​ litter ​​ ​​ waning ​​ ​​ weight−total ​​ ​​ starting ​​ ​​ litter ​​ ​​ weight+removal ​​ ​​ weights)/lactation ​​ ​​ length. Piglet removal rate was calculated as the number of mortalities plus morbidities divided by litter size after cross-fostering.

### Sample collection and analysis

#### Diet analysis

The weekly feed samples for each dietary treatment were pooled on an equal weight basis and ground using a Wiley Mill (Variable Speed Digital ED-5 Wiley Mill; Thomas Scientific, Swedesboro, NJ) through a 1 mm screen. The ground samples were submitted to the University of Missouri Agricultural Experimental Station Laboratories (Columbia, MO) for complete amino acid profiling using cation-exchange chromatography coupled with postcolumn ninhydrin derivatization and quantification (method 982.0 E and 988.15; AOAC, 2006). The diet samples were also analyzed in duplicate for gross energy using an isoperibolic bomb calorimeter (model 6200; Parr Instrument Co., Moline, IL). Benzoic acid (6,318 kcal/kg) was used as the standard for calibration and was determined to contain 6,322 ± 1.3 kcal/kg.

#### Milk sample collection and analysis

At the time of weaning, approximately 50 mL of milk was collected from 20 sows per treatment (four sows per lactation group). For milk sample collection, 1.5 mL of oxytocin was administered intramuscularly near the vulva approximately 1 h after piglet removal, and a clean gloved hand was used to expel milk from the second through sixth anterior glands on the left side of the sow. Within a lactation group, sows for milk sample collection were chosen to minimize the differences in lactation length. After collection, the milk samples were immediately placed on ice and stored at −80 °C until analysis. Milk samples were submitted to CentralStar Labs (Grand Ledge, MI) for composition analysis of fat, true protein, lactose, and milk urea nitrogen (**MUN**) using Fourier-transformed infrared spectroscopy (Zhang et al., 2019).

### Statistical analysis

Data were analyzed in R (v4.4.1; R Core Team, 2024) using generalized linear mixed models with fixed effects of dietary treatment, parity group (P < 3 or P3+), and their interaction and a random effect of lactation group. Calculated postfarrowing sow BW and entry BF were fit as covariates in the models for sow BW and BF change, respectively. For litter performance, number of piglets after cross-fostering was fit as a covariate for number weaned, litter weight after cross-fostering was fit as a covariate for litter weaning weight and litter average daily gain (**ADG**), and average piglet starting BW was fit as a covariate for average piglet weaning weight and ADG.

The models for sow feed intake, BW, BF, ME balance, litter growth rate, and milk composition were fit assuming a normal distribution using the lmer function from the lme4 package (v1.1.35.3; Bates et al., 2015). The models for lactation length, WEI, litter counts, and piglet removals were fit using the glmer function from the lme4 package, assuming a Poisson distribution for lactation length, WEI, and litter counts and a binomial distribution for piglet removals. The model for sow parity was fit assuming a negative binomial distribution using the glmer.nb function from the MASS package (v7.3.60.2; Venables and Ripley, 2002).

The assumptions of all models were verified through residual diagnostic plots created with the check_model function from the performance package (v0.12.0; Lüdecke et al., 2021). For models assuming a normal distribution, Studentized Residuals greater than approximately three standard deviations from the mean were considered statistical outliers and excluded from the analysis. Data are reported as least squares means and means separation was conducted using the emmeans package (v1.10.2; Lenth, 2024) with Tukey adjustment for multiplicity. The sow and her litter were the experimental unit; results were considered significant if *P* ≤ 0.05.

## Results

The chemical analysis of the experimental diets indicated that the total amino acid concentrations were consistent with calculated values based on expected analytical variation (Table 2; Cromwell et al., 1999). Additionally, the analyzed CP values were consistent with formulated values with LSBM and LSBM + BCAA diets having lower CP than HSBM and HSBM + BCAA. Furthermore, similar analyzed gross energy values across diets supported the isocaloric formulation strategy.

**Table 2.** Analyzed nutrient composition of experimental diets (as-fed basis)

	Dietary treatment			
Item	LSBM	HSBM	LSBM + BCAA	HSBM + BCAA
Gross energy, Mcal/kg	3.83	3.93	3.85	3.90
Crude protein, %	16.39	21.34	17.64	19.65
Total amino acids, %				
Alanine	0.86	1.06	0.79	0.97
Arginine	1.01	1.40	0.89	1.25
Aspartic Acid	1.58	2.21	1.38	1.96
Cysteine	0.27	0.33	0.23	0.30
Glutamic Acid	2.97	3.95	2.65	3.53
Glycine	0.67	0.89	0.60	0.80
Histidine	0.43	0.56	0.38	0.51
Isoleucine	0.69	0.95	0.80	0.84
Leucine	1.45	1.83	1.63	1.81
Lysine	1.18	1.28	1.06	1.13
Methionine	0.25	0.30	0.22	0.28
Phenylalanine	0.81	1.08	0.72	0.97
Proline	0.99	1.23	0.91	1.13
Serine	0.73	0.94	0.65	0.86
TBCAA	3.12	3.84	3.32	4.02
Threonine	0.76	0.86	0.74	0.77
Tryptophan	0.28	0.26	0.24	0.24
Tyrosine	0.58	0.76	0.52	0.68
Valine	0.98	1.06	0.89	1.37

The average parity across treatments was 3.2 (Table 3; Trt *P* = 0.997), and, on average, sows nursed their litters for 19.7 days (Trt *P* = 0.972). There was no evidence for a difference in calculated sow BW after farrowing (Trt *P* = 0.328), with a range between treatments in calculated 48 h postfarrowing BW of 207 to 212 kg. On average, sows gained 7.1 kg (Trt *P* = 0.682) or 3.4% of their BW through lactation. The calculated change in sow BW was reflected in calculated ME balance, where sow ME intake relative to the estimated requirements for maintenance and milk energy secretion was similar across treatments (Table 4; Trt *P* = 0.271). Similarly, there was little evidence that sow BF differed at the time of entry into the farrowing house (Trt *P* = 0.102), and sows lost an average of 1.3 mm (Trt *P* = 0.928) or 12.7% BF. Average daily feed intake (**ADFI**) was reduced in sows fed HSBM compared to LSBM (7.05 vs. 7.43 kg/d; *P* = 0.024), while sows fed LSBM + BCAA had similar ADFI to LSBM (7.24 vs. 7.43 kg/d; *P* = 0.515) or HSBM (*P* = 0.466). Furthermore, sows fed HSBM + BCAA exhibited reduced feed intake compared to LSBM (6.87 vs. 7.43 kg/d; *P* < 0.001) and LSBM + BCAA (*P* = 0.025), but similar feed intake to HSBM-fed sows (*P* = 0.512).

**Table 3.** Effect of dietary treatments on sow lactation and subsequent reproductive performance

	Dietary treatment					*P*-value		
Item	LSBM	HSBM	LSBM + BCAA	HSBM + BCAA	SEM^1^	Trt	Parity	Trt × Parity
Number of sows, n	89	87	88	88	–	–	–	–
Average parity	3.2	3.2	3.2	3.2	0.32	0.997	–	–
Sow ADFI, kg	7.43^a^	7.05^bc^	7.24^ab^	6.87^c^	0.095	<0.001	<0.001	0.321
Sow BW, kg								
Calculated 48 h postfarrowing	212	207	210	210	2.8	0.328	<0.001	0.420
Change (48 h—weaning)^2^	8.3	7.7	6.5	5.9	1.64	0.682	<0.001	0.191
Sow BF, mm								
Entry	10.4	9.7	10.4	10.6	0.27	0.102	0.949	0.204
Change (entry—weaning)^3^	−1.3	−1.4	−1.2	−1.3	0.13	0.928	<0.001	0.122
Lactation length, d	19.7	19.6	19.7	19.9	0.48	0.972	0.839	0.999
Wean-to-estrus interval, d	4.6	4.8	4.6	4.9	0.26	0.797	0.039	0.763
Subsequent performance								
Number of sows, n	78	75	79	74	–	–	–	–
Farrowing rate, %^4^	92.1	91.2	95.9	84.3	5.32	0.132	0.449	0.419
Subsequent total born, n/sow	17.1	16.7	16.4	17.4	0.56	0.559	0.225	0.052
Subsequent born alive, n/sow	14.7	14.2	14.3	14.6	0.56	0.897	0.082	0.198

^1^Maximum value of the SE of the least squares means.

^2^Calculated sow 48 h postfarrowing weight included as covariate (*P* < 0.001).

^3^Entry BF included as covariate (*P* < 0.001).

^4^Farrowing rate = number of sows bred by day 14/ number of sows farrowed.

^a-c^Within a row, means without a common superscript differ (*P* ≤ 0.05).

**Table 4.** Calculated ME balance of sows during lactation

	Dietary treatment					*P*-value		
Item	LSBM	HSBM	LSBM + BCAA	HSBM + BCAA	SEM^1^	Trt	Parity	Trt × Parity
ME intake, Mcal/d	23.77^a^	22.56^bc^	23.18^ab^	21.97^c^	0.303	<0.001	<0.001	0.321
ME_m_, Mcal/d	6.10	5.99	6.05	6.06	0.060	0.343	<0.001	0.451
ME_l_, Mcal/d	16.99	16.25	17.09	16.10	0.379	0.003	0.001	0.017
ME balance, Mcal/d	0.74	0.45	0.05	-0.07	0.395	0.271	<0.001	0.229

^1^Maximum value of the SE of the least squares means.

^a-c^Within a row, means without a common superscript differ (*P* ≤ 0.05).

The average WEI across treatments was 4.7 days (Table 3; Trt *P *= 0.797). Farrowing rate did not differ between treatments (Trt *P* = 0.132), and there was no evidence for differences in subsequent total born (Trt *P* = 0.559) or number born alive (Trt *P* = 0.897).

After cross-fostering, sows started the trial with an average of 14.0 piglets/sow (Table 5; Trt *P* = 0.787) with an average litter weight of 20.36 kg (Trt *P* = 0.535). Consequently, average piglet start weight was similar across treatments (Trt *P* = 0.487). On average, sows weaned 12.6 piglets/sow (Trt *P* = 0.875) with a range between treatments of 12.4 to 12.8 piglets weaned per sow. There was an interaction between treatment and parity for litter weaning weight (Trt × Parity *P* = 0.022), where numerical differences in number weaned resulted in sows fed LSBM + BCAA weaning heavier litters compared to HSBM sows (80.27 vs. 73.77 kg; *P* = 0.001) or HSBM + BCAA-fed sows (80.27 vs. 73.99 kg; *P* = 0.001), while litters of sows fed LSBM were intermediate in weight at weighing. There were no differences in litter weaning weight in parity 1 and 2 sows. Consequently, a similar interaction was observed for litter average daily gain (ADG; Trt × Parity *P* = 0.023), where litter ADG was higher in parity 3 + sows fed LSBM + BCAA compared to sows fed HSBM (3.12 vs. 2.88 kg/d; *P* = 0.005) or HSBM + BCAA (3.12 vs. 2.79 kg/d; *P* < 0.001). However, there was no evidence for a difference in individual piglet ADG across treatments (Trt *P* = 0.646). Furthermore, although numerically lowest in litters of LSBM-fed sows, there is little statistical evidence to suggest piglet removals differed between treatments (Trt *P* = 0.143).

**Table 5.** Effect of dietary treatments on litter performance

	Dietary treatment					*P*-value		
Item	LSBM	HSBM	LSBM + BCAA	HSBM + BCAA	SEM^1^	Trt	Parity	Trt × Parity
Number started, n/sow	14.1	14.1	14.0	13.6	0.40	0.787	0.076	0.958
Litter start weight, kg	20.75	20.45	20.11	20.11	0.399	0.535	0.202	0.549
Average piglet start weight, kg	1.48	1.46	1.44	1.48	0.024	0.487	<0.001	0.702
Number weaned, n/sow^2^	12.8	12.4	12.7	12.5	0.38	0.875	0.139	0.996
Litter weaning weight, kg^3^	76.12	73.08	76.90	74.0	1.084	0.004	0.007	0.022
Average piglet weaning weight, kg^4^	5.93	5.88	5.98	5.96	0.083	0.633	<0.001	0.228
Litter ADG, kg/d^3^	2.93	2.81	2.96	2.80	0.053	0.001	<0.001	0.023
Piglet ADG, kg/d^4^	0.22	0.22	0.22	0.22	0.004	0.646	<0.001	0.338
Removals, %	7.90	10.62	9.05	9.31	0.925	0.143	0.002	0.804

^1^Maximum value of the SE of the least squares means.

^2^Number started included as covariate (*P* < 0.001).

^3^Litter start weight included as covariate (*P* < 0.001).

^4^Average piglet start weight included as covariate (*P* < 0.001).

Milk composition analysis indicated that the dietary treatments affected true protein (Table 6; Trt *P* = 0.012) and MUN (Trt *P* < 0.001) but not fat (Trt *P* = 0.249) or lactose (Trt *P* = 0.764) concentration. Milk true protein concentration was lower in sows fed HSBM compared to LSBM + BCAA (4.49 vs. 4.80%; *P* = 0.016) or HSBM + BCAA (4.49 vs. 4.77%; *P* = 0.026), while milk from sows fed LSBM was at an intermediate level. Furthermore, MUN was lower in sows fed LSBM compared to HSBM (25.9 vs. 32.4 mg/dL; *P* < 0.001) or HSBM + BCAA (25.9 vs. 32.3 mg/dL; *P* < 0.001), and at an intermediate level in sows fed LSBM + BCAA.

**Table 6.** Effect of dietary treatments on sow milk composition at weaning

	Dietary treatment					*P*-value		
Item	LSBM	HSBM	LSBM + BCAA	HSBM + BCAA	SEM^1^	Trt	Parity	Trt × Parity
Fat, %	6.74	7.05	6.49	6.73	0.221	0.249	0.397	0.244
True protein, %	4.71^ab^	4.49^b^	4.80^a^	4.77^a^	0.086	0.012	0.260	0.997
Lactose, %	5.41	5.38	5.45	5.40	0.048	0.764	0.848	0.321
Milk urea N, mg/dL	25.9^b^	32.4^a^	29.0^ab^	32.3^a^	1.216	<0.001	0.804	0.388

^1^Maximum value of the SE of the least squares means.

^a-b^Within a row, means without a common superscript differ (*P* ≤ 0.05).

## Discussion

In the current experiment, lactation ADFI was reduced by approximately 5% (7.43 vs. 7.05 kg/d) when SBM inclusion in the diet was increased from 23.3% to 37.3%, which resulted in dietary CP levels of 16.7% and 21.3% in the LSBM and HSBM diets, respectively. Similarly, Gourley et al. (2020) reported an approximate 9% reduction in ADFI (5.7 vs. 5.2 kg/d) in response to increasing SBM inclusion from 25% to 35% (18 vs. 22% CP) in sow lactation diets. In both the current experiment and that of Gourley et al. (2020), feed-grade amino acids were included as needed to maintain constant Lys, Met + Cys, Thr, Trp, and Val levels, while the other essential and nonessential amino acids increased with increasing SBM inclusion. Reductions in sow feed intake have also been reported in studies evaluating increasing Lys through the addition of SBM in the diet, suggesting factors other than Lys are impacting sow ADFI with increased SBM in the diet (Yang et al., 2000a; Gourley et al., 2017). For example, Yang et al. (2000a) increased total Lys from 0.60% to 1.60% by increasing SBM from 14.5% to 48.5% and CP from 13.6% to 28.8% and reported a linear reduction in sow ADFI from 5.4 to 4.6 kg/d. Recently, Estrada et al. (2024) observed a 4.3% reduction in feed intake in third to fifth parity sows when SID Lys increased from 0.85% to 1.11%, which was achieved by increasing SBM, but no effect in first and second parity sows. In contrast, other researchers have reported no impacts of increasing SBM inclusion above 30% on sow ADFI with either a constant (Greiner et al., 2018) or increasing SID Lys supply (Touchette et al., 1998). However, notable differences in these studies compared to the current study are that sows were limit-fed to control maximal Lys intake in Greiner et al. (2018), and sows in Touchette et al. (1998) exhibited lower feed intake than commonly observed in modern swine production (PIC North America, 2024). Therefore, restricted feed intake in studies that have not observed a reduction in ADFI with increasing SBM inclusion may have masked differences from being detected. Additionally, previous work evaluating dietary CP level in lactating sows has reported no impact of increasing CP on sow ADFI (Hojgaard et al., 2019a, 2019b); however, the dietary SBM levels ranged from 12.0% to 26.1% in Hojgaard et al. (2019a) and 2.7% to 23.7% in Hojgaard et al. (2019b), suggesting SBM inclusion may not have been high enough to adversely affect feed intake in those studies.

In the current study, the relative balance of BCAA was adjusted in the LSBM + BCAA and HSBM + BCAA diets based on previous suggestions that imbalances between the BCAA associated with increased SBM inclusion may cause reductions in sow feed intake (Yang et al., 2000a; Gourley et al., 2020). The first two steps in the pathway of BCAA catabolism are shared amongst the three amino acids, which include transamination by branched-chain amino acid transferase and subsequent decarboxylation by the branched-chain *α*-keto acid dehydrogenase (BCKD) enzyme complex. The flux-generating of the BCAA catabolic pathway is irreversible decarboxylation by BCKD, which is covalently and allosterically regulated. Accordingly, increased BCKA concentration caused by excess BCAA will increase flux through the BCAA catabolic pathway. The lockstep catabolism of Leu, Ile, and Val is the biological mechanism behind the commonly described BCAA antagonism, whereby excess of one BCAA will stimulate the catabolism of all three amino acids, which can lead to deficiency of the BCAA not in excess (D’Mello, 2003). Consequently, amino acid-sensing regions of the brain signal the animal to reduce feed intake, which is a consistent phenotype associated with amino acid deficiency or imbalance (Harper et al., 1970; Heeley and Blouet, 2016). Therefore, balancing the SID Ile:Leu:Val levels in HSBM + BCAA relative to the LSBM diet should have mitigated potential issues associated with an antagonism due to BCAA imbalance, preventing a subsequent decline in feed intake.

In contrast to the hypothesis, sows consuming either HSBM or HSBM + BCAA exhibited reduced feed intake compared to sows fed LSBM, indicating that adjusting the relative balance of BCAA did not prevent the decline in feed intake associated with increasing SBM in the diet. Interestingly, sows fed HSBM + BCAA had the lowest numerical level of feed intake, although there was little statistical evidence to suggest intake was lower than sows fed HSBM. Furthermore, feed intake in sows fed LSBM + BCAA was slightly reduced, resulting in an intermediate level of feed intake compared to LSBM and HSBM, but again, this numerical reduction did not result in statistical differences between LSBM + BCAA and LSBM or HSBM. Together, this provides little evidence to suggest that BCAA balance contributed to the observed decrease in sow lactation feed intake with increasing SBM in the diet.

Alternatively, BCAA may influence feed intake by indirectly interfering with neurotransmitter biosynthesis through competitive inhibition of large-neutral amino acid (**LNAA**) uptake at the blood–brain barrier. Branched-chain amino acid transport across the blood–brain barrier is facilitated primarily by the L-system transporters, which are saturable and shared by other LNAA. Of the LNAA, the aromatic amino acids Trp, Phe, and Tyr are precursors of the feed intake-related neurotransmitters serotonin, dopamine, and norepinephrine (Fernstrom, 2005).

In the present study, increasing SBM inclusion resulted in SID total BCAA (TBCAA) increasing from 2.82% in LSBM to 3.42% in HSBM, and adjusting the BCAA balance in HSBM + BCAA resulted in a SID TBCAA level of 4.00%. Similarly, SID Leu concentration increased from 1.27% in the LSBM to 1.61% in HSBM and 1.80% in HSBM + BCAA, and SID Ile and Val concentrations increased from 0.62% to 0.87% and 0.93% to 1.33%, respectively. Therefore, increasing concentrations of BCAA, rather than imbalances, may have contributed to the observed reduction in sow ADFI; however, recent research conducted by the authors evaluated greater ranges and levels of TBCAA, Leu, and Val, suggesting this may not be the case (Humphrey et al., 2024). Additionally, with increased SBM inclusion in HSBM and HSBM + BCAA, SID Phe and Tyr increased by 0.24% and 0.14% of the diet, respectively, while SID Trp was constant in all diets. Although there has been no work investigating the effect of BCAA relative to Phe and Tyr on swine feed intake, reduced circulating serotonin due to impaired Trp uptake is a proposed mechanism for decreased feed intake in growing pigs associated with excess dietary Leu (Cemin et al., 2019; Kwon et al., 2019). Furthermore, Trottier and Easter (1995) reported decreased feed intake in lactating sows when dietary total Trp:TBCAA decreased from 6.4% to 4.1% through the addition of BCAA to the diet. In the current study, SID Trp:TBCAA decreased from 8.6% in LSBM to 7.1% in HSBM and LSBM + BCAA and 6.1% in HSBM + BCAA. Therefore, if competition with other LNAA is a mechanism by which BCAA are contributing to reduced feed intake with elevated SBM, adjusting the balance of BCAA in the HSBM diet would not be expected to mitigate the response.

Although not measured in the current study, SBM contains several antinutritional factors, such as trypsin inhibitors (TI), lectin, raffinose, and stachyose. Specifically, TI may reduce feed intake through cholecystokinin-mediated mechanisms (Woyengo et al., 2017). Previous work with raw soybeans, which contain higher TIU levels than SBM, supports the concept that lactating sow feed intake may be affected by TI (Yen et al., 1991). Woyengo et al. (2017) suggested that pigs can tolerate up to 3.00 TI units (TIU)/mg. García-Rebollar et al. (2016) analyzed the TI activity in 180 U.S. soybean meal samples and reported a range in TI activity of 1.4 to 5.5 mg/g dry matter. Therefore, even if the TIU level in the SBM from the current study was at the upper end of this range, it is unlikely the concentration of TIU in the HSBM and HSBM+BCAA diets reached the threshold suggested by Woyengo et al. (2017). However, to the authors’ knowledge, there is no published literature directly evaluating the impacts of TI on lactating sow performance; thus, further work is warranted to understand if these antinutritional factors are related to reduced feed intake with increased SBM in the diet.

Alternatively, the presence of undigested nutrients in the small intestine and colon may stimulate intestinal brake mechanisms to reduce gastric emptying, rate of passage of digesta through the small intestine, and feed intake (Black et al., 2009). While there were only small differences in calculated concentrations of neutral detergent fiber and acid detergent fiber across dietary treatments, CP increased by approximately 4.5% with increased SBM inclusion. Consequently, greater amounts of undigested protein entering the distal small intestine and colon as a result of increased SBM inclusion could have reduced gastrointestinal motility and increased digesta transit time, resulting in feedback signals to reduce meal size and frequency. In addition, the stimulation of intestinal break mechanisms leads to the release of anorexigenic peptides from the gastrointestinal tract, such as peptide tyrosine tyrosine, glucagon-like peptide-1, oxyntomodulin, and apolipoprotein A-IV, which contribute to the long-term control of feed intake (Black et al., 2009). Previous researchers evaluating dietary CP level in lactation have not reported reduced sow ADFI with increasing CP up to 18.1% (Dourmad et al., 1998; Strathe et al., 2017; Hojgaard et al., 2019a, 2019b); However, the CP levels evaluated in the current study and those of Gourley et al. (2020) and Yang et al. (2000a) were much higher, ranging from 14.7% to 28.8%. Therefore, the combined short- and long-term feed intake-regulating mechanisms stimulated by increased dietary CP may have contributed to the observed reduction in sow ADFI with increased dietary SBM inclusion.

In contrast to previous research, the reduction in ADFI associated with increasing SBM inclusion in the current study was not accompanied by increased calculated sow BW or BF losses in lactation. The average ME intake across treatments ranged from 22.0 to 23.8 Mcal/d. Based on the calculated ME requirements for maintenance and energy secreted in milk, the observed levels of ME intake resulted in an estimated ME balance ranging from −0.07 to 0.74 Mcal/d, suggesting sows were near cumulative energy balance through lactation. Consistent with ME balance, although sow ADFI differed in response to dietary SBM inclusion, variation in LGR resulted in similar sow feed efficiency, where, on average, one kg of sow feed intake resulted in approximately 0.40 kg of litter gain across treatments. Additionally, the calculated ME balances for each treatment aligned with the observed magnitude of calculated BW gain in lactation. Although sows gained weight in lactation, aside from LSBM-fed sows, the magnitude of increase was less than a single day’s feed intake, which may indicate the gain in BW was reflective of gut fill at the time of weaning rather than actual tissue accretion. Furthermore, the average total Lys intake ranged from 89 to 92 g/d, which is greater than the calculated total Lys requirements of 67 to 71 g/d based on Greiner et al. (2020). Together, this suggests that, across treatments, sows in the current study were near cumulative energy and Lys balance through lactation, which explains the lack of response of BW to differences in sow feed intake with increased SBM inclusion.

In contrast, sows fed the lowest level of SBM in Gourley et al. (2020) would have been in a negative ME balance of approximately 4.7 Mcal/d, which was further increased to nearly 6.7 Mcal/d with the reduced feed intake reported in sows fed the diet containing 35% SBM. Additionally, total Lys intake ranged from 62 to 67 g/d, which is below the calculated requirement of 70 to 73 g/d (Greiner et al., 2020). Consequently, the lower level of feed intake observed across all sows in Gourley et al. (2020) compared to the sows in the current study may explain the differing response of sow BW loss to increased SBM in the diet.

In the current study, litter weaning weight was increased in parity 3 + sows fed LSBM + BCAA compared to HSBM or HSBM + BCAA; however, these differences were not reflected in individual piglet weaning weight, suggesting numerical differences in number of piglets started and weaned were driving the observed differences in litter growth in these sows. Interestingly, there was no evidence that individual piglet growth rate differed across the dietary treatments, despite differences in true protein concentration in milk, which suggests the differences in true protein concentration did not correspond to differences in total true protein yield or that differences were not of great enough magnitude to induce alterations in piglet growth. Furthermore, MUN increased with dietary CP concentration, reflecting the expected rise in circulating urea associated with higher CP intake (Roseler et al., 1993; Yang et al., 2000b; Gourley et al., 2020). It is well known that blood urea nitrogen and MUN are strongly correlated (Roseler et al., 1993; Burgos et al., 2007; Prahl et al., 2023). Thus, an oversupply of dietary CP increases circulating urea concentration, leading to a corresponding rise in MUN. Since all diets in the current study provided adequate essential amino acids according to NRC (2012), increasing SBM resulted in an excess of certain essential amino acids and a surplus of nonessential amino acids, leading to greater urea export in milk. Therefore, the results of the current study suggest that MUN may be used as an indicator of dietary CP oversupply in sows.

In conclusion, while adjusting BCAA in the LSBM diet slightly reduced sow feed intake compared to the LSBM diet, balancing BCAA in the HSBM diet did not alleviate the reduction in ADFI caused by elevated SBM. This suggests that factors beyond BCAA balance are contributing to reduced feed intake associated with elevated SBM in sow lactation diets, emphasizing the need for further research into other dietary components or physiological mechanisms that may be involved.

## Abbreviations:

ADFI: average daily feed intake
ADG: average daily gain
BCAA: branched-chain amino acids
BCKD: branched-chain α-keto acid dehydrogenase
BCS: body condition score
BW: body weight
CP: crude protein
LNAA: large-neutral amino acid
ME: metabolizable energy
ME_l_: metabolizable energy required for energy secreted in milk
ME_m_: metabolizable energy requirement for maintenance
MUN: milk urea nitrogen
NIR: near-infrared spectroscopy
SBM: soybean meal
SID: standardized ileal digestible
TBCAA: total branched-chain amino acids
TI: trypsin inhibitor
TIU: trypsin inhibitor unit
WEI: wean-to-estrus interval
